# Randomized trials of alcohol-use interventions with college students and their parents: lessons from the Transitions Project

**DOI:** 10.1177/1740774510396387

**Published:** 2011-04

**Authors:** AC Fernandez, MD Wood, R Laforge, JT Black

**Affiliations:** Department of Psychology, University of Rhode Island, Kingston, RI, USA

## Abstract

***Background*** Matriculation from high school to college is typified by an increase in alcohol use and related harm for many students. Therefore, this transition period is an ideal time for preventive interventions to target alcohol use and related problems.

***Purpose*** The purpose of this report is to describe the design and methods used in the Transitions Project, a randomized controlled trial of two interventions designed to prevent and reduce heavy episodic drinking and alcohol-related negative consequences among incoming college students.

***Methods*** This study used a 2 × 2 factorial design to investigate the effects of a two-session brief motivational intervention delivered to students and a handbook-based parent intervention. Interventions were administered to students and parents. Follow-up assessment took place at 10- and 22-months post-baseline.

***Results*** The Transitions Project successfully recruited and retained participants across a major transition period (i.e., entering college), administered and compared two distinct but complementary interventions, and collected and analyzed highly skewed data. The application of a factorial design and two-part latent growth curve modeling allowed us to examine main and interactive intervention effects in terms of both initiation and growth in heavy drinking and alcohol-related problems.

***Limitations*** While we conducted successful tests of our primary and secondary study hypotheses over a lengthy follow-up period, our study design did not permit full interpretation of null findings. We suggest that researchers carefully consider assessment timing, tests of assessment reactivity, and ensure objective tests of intervention efficacy when conducting clinical trials of motivational interventions.

***Conclusions*** The lessons we learned while conducting this trial have the potential to assist other researchers designing and conducting future preventive interventions targeting parents and college students. The data analytic procedures presented can also help guide trials that plan to analyze zero-inflated non-normal outcome data.

## Background

The Transitions Project was a randomized controlled trial designed to examine the unique and combined effects of two preventive interventions to reduce heavy episodic drinking (HED) and related-harm among incoming college students. The purpose of this report is to describe the methodology of this trial and share the lessons learned in terms of recruiting and retaining a large sample across a major transition period (i.e., entering college), administering and comparing two complementary interventions, and collecting and analyzing zero-inflated non-normal data using a novel data-analytic technique. The reduction of HED among college students is a national priority [1,2]. Over 70% of college students report using alcohol, and approximately 40% report HED (typically defined as five drinks in a row for men and four for women) [3]. While alcohol use is common among late adolescents, the passage from high school to college is typified by an increase in alcohol consumption and associated negative consequences [4–6]. Thus, the transition from high school to college represents a period of critical importance with respect to preventing and reducing heavy drinking and alcohol-related negative consequences.

Brief motivational interventions are one-on-one counseling sessions that utilize motivational interviewing and personalized feedback to facilitate change in health-related behaviors. They are the most empirically supported individual-level intervention for reducing alcohol use and problems among heavy drinking college students [7–13]. We expanded on past research by administering the brief motivational intervention to college students with heterogeneous drinking experiences and extending assessment periods beyond 1 year.

Parent-based interventions are an emerging approach to prevent college student drinking. These interventions target parents and promote parenting strategies associated with lower levels of alcohol use and problems in adolescence; they build on research indicating that parents exert a continued influence throughout the college years [14–17]. However, the potential beneficial role of parental influence on college student drinking has not been widely investigated [18–22], despite a substantial body of research documenting the efficacy of family and parent-based substance use interventions for younger adolescents [23–26].

### This study

The Transitions Project was designed to simultaneously examine a brief motivational and parent-based intervention as a means to reduce the onset and growth of college student HED and alcohol-related consequences. A factorial design was chosen to examine complementary influences on college student drinking (parent and peer factors) [27] and to attempt to increase effect sizes and reduce cost by combining interventions in one stand-alone trial [10,28].

## Method

The Transitions Project used a 2 × 2 × 3 design with two dichotomous between-subjects factors, brief motivational intervention (yes, no) and parent-based intervention (yes, no), and one within-subjects factor (Time; pre-matriculation, 10 months, 22 months). It was hypothesized that relative to an assessment only control arm, the study interventions would reduce the initiation and growth of HED and consequences among incoming college students, and the intervention effect would be multiplicative; (i.e., combined intervention effects greater than the sum of the individual effects). Secondary aims of this study included tests of intervention mediators such as changes in descriptive norms, for the brief motivational intervention, and changes in parental monitoring, for the parent-based intervention.

### Recruitment and retention

Eligible students and their parents were recruited from two successive cohorts of incoming students at a mid-sized northeastern public university in the United States. All procedures were approved and monitored by the university Institutional Review Board (IRB).

#### Eligibility criteria

The target population for this research trial was matriculating first year students ages 17–21 and their parents. Non-traditional students (e.g., older, married, returning, and transfer students) were not eligible to participate because of the emphasis on parent communication within the home. Biological parents, stepparents, and legal guardians were eligible to participate as long as they were living with the student during the recruitment period.

#### Recruitment and consent

Recruitment took place by telephone in the summer prior to college matriculation. Prior to the first telephone contact, a detailed consent form and introductory letter were mailed to potential participants. For students who were under 18 years of age during the recruitment phase, an assent form as well as a parental permission letter was sent in lieu of the standard consent form. Upon telephone contact, interested parents and students (ages 18 and older) were asked to provide oral consent and complete the baseline assessment over the telephone. Students who were 17 years old were required to provide oral assent and parental consent over the telephone. In-person consent was waived because recruitment took place before the students arrived on campus. IRB approval for oral consent was obtained in accordance with 45 CFR 46 ‘Protection of Human Subjects,’ Section 46.117 c.2.

Given the power differential between parent and child, we chose to recruit students prior to parents to minimize intentional or unintentional parental pressure. All participants were informed that their participation was voluntary, and students were paid for their baseline participation regardless of parent recruitment. Confidentiality procedures did not guarantee complete anonymity for study participation due to recruitment of family members within the same household.

In an effort to recruit a gender-balanced parent sample, a mother or father was randomly chosen as the initial recruitment target. If the pre-determined parent was unwilling or unavailable, another parent was accepted for recruitment (regardless of gender). We took this approach because fathers have been under-represented in parent-based alcohol interventions with college students [14,27,29,30]. All data were collected through a professional survey center which utilized computer-assisted telephone interviewing. Interviewers were trained, certified, and monitored periodically in the proper application of standardized interviewing procedures and study protocols [31]. Interviewers were blind to study arms.

#### Randomization and retention

Student–parent dyads were randomized to treatment arms after consenting and completing the baseline assessment. Our trial used standard protocols for subject tracking and multiple attempts to contact participants in each follow-up period regardless of university enrollment status or participation in previous assessments [32]. Home and local contact information was collected at baseline and confirmed at all time points. Two supplementary contacts, i.e., people who would know the participants’ whereabouts at all times, were collected at each time point.

#### Incentives

Students received $30 for completing the baseline interview, $40 for completing the 10-month follow-up procedures, and $50 after completing the 22-month follow-up procedures. Participants who attended all appointments and completed follow-up procedures on time were eligible for $10 and $20 cash bonuses at the 10- and 22-month follow-ups, respectively. Three $200 cash prizes were awarded annually to randomly selected student participants. Parents were offered $40 at each time point.

### Assessments

Student follow-ups took place at 10 and 22 months and were anchored according to the baseline completion date. Assessment time points were chosen to assess students while they were on campus and capture long-term (>6 months) intervention effects rarely examined in past research. The parent follow-up took place at 12 months post-baseline and was timed to coincide with students return home for their first summer break in order to adequately assess parenting behaviors. All student assessments took place by telephone and lasted approximately 45–60 min. Parent follow-ups took place by mail.

### Interventions

#### Brief motivational intervention

The brief motivational intervention used in this study was modeled after the Brief Alcohol Screening and Intervention for College Students (BASICS) program [33] and included two counselor-facilitated in-person interventions during the freshman year. The initial 1-h meeting took place in the fall, and the half-hour ‘booster’ session took place in the spring. Counselors (*n* = 16) were bachelor’s- and master’s-level psychology students trained in motivational interviewing and intervention content. Training and weekly group supervision were conducted by a PhD-level psychologist with years of experience in delivering, supervising, and researching brief motivation interventions [11].

A central component of the brief motivational intervention is the delivery of ‘personalized feedback’ to students regarding alcohol-related behavior and beliefs. Feedback forms were created using assessment data gathered approximately 2 weeks prior to scheduled interventions (once in the fall and once in the spring). Drinkers received feedback on their current alcohol use and related-problems as well as their drinking compared to ‘average students.’ Abstainers received feedback on the safety and health benefits of abstinence, their experiences with second-hand effects of alcohol, and their abstinence-related self-efficacy. Booster sessions incorporated feedback on current and past drinking to reflect change in alcohol-related behavior since the initial evaluation. Clinicians were trained to present all feedback using an empathic non-confrontational style and students were given feedback forms to take home.

To monitor intervention fidelity, the clinical supervisor randomly selected 5–10% of audio tapes (50 for the initial fall intervention and 26 for the spring intervention), and coded them in their entirety using the Motivational Interviewing Treatment Integrity scale [34]. Written feedback was provided to counselors. Session evaluation forms were also completed by students and counselors after each session to assess the quality and delivery of intervention components. Evaluation forms were similar across time points.

#### Parent-based intervention

The parent-based intervention consisted of a 32-page parent handbook which was mailed to parents in the summer before students matriculated to college. The handbook itself was modified from an original version [30] and included information designed to raise parental awareness of college student alcohol use and provide strategies to help reduce student drinking and associated consequences. These strategies included increasing parent–teen alcohol-related communication and parental monitoring, and reducing parental permissiveness for drinking. A ‘booster’ letter was mailed a year later that reviewed handbook concepts and encouraged parents to continue to implement strategies to reduce college student alcohol abuse.

In addition to the parent handbook, parents received a letter explaining the intervention, and a handbook evaluation form. The letter informed parents that their evaluations were needed to assess and improve the handbook [30]. We hoped that this letter would encourage all parents to read the materials and provide us with feedback. The evaluation form served as our primary measure of intervention fidelity and assessed readability, usefulness, and clarity of the handbook. Parents who did not return the evaluation by mail were contacted *via* telephone by the survey research center.

### Outcomes and data analysis

At all time points, students were assessed regarding: (1) whether and how frequently they engaged in HED; (2) whether and how often they experienced alcohol-related negative consequences; and (3) hypothesized intervention mediators. Because our study was designed to enroll students with a range of alcohol-related experiences, our data contained a large proportion of zero values (i.e., students who did not drink) in addition to data reflecting very heavy drinking. To address this skew in our data distribution, we chose to conduct our analysis using two-part latent growth curve-modeling. This technique is well suited to address the heterogeneity arising from zero-inflated data by simultaneously creating two correlated models from a single outcome variable; one model for the binary (onset) portion and one model for continuous (rate of change) portion of the variable’s distribution [35,36]. In Part 1 (the binary portion), the outcome variable is modeled as a random-effects logistic growth model with the log odds of use regressed on growth factors [37]. In Part 2 (the continuous portion), the non-zero continuous frequency of the outcome is modeled using the latent growth model [38]. For this study, the binary part of the model estimated growth in onset of HED or consequences (coded as 0 and 1). The continuous part of the model estimated change in the frequency of HED or consequences for drinkers who initially reported one or more instances of these behaviors.

An important advantage of the two-part model approach over the censored normal model [39] for fitting discrete mixture models to longitudinal zero-inflated data [39,40] is the ability to estimate the unique effects of covariates on each of the two parts even when they are correlated [37,38]. Two-part latent growth models, therefore, enable the separate evaluation of intervention, mediating, and covarying factors on onset and growth of outcome variables. Thus, we were able to determine whether our interventions affected mediators and whether the mediators affected change in onset and growth of outcome variables. We estimated the effects of the brief motivational intervention, the parent-based intervention, and their interaction on all 10-month mediators regardless of an overall intervention effect to determine whether the intervention significantly changed the hypothesized mediator(s) in the desired direction and whether the mediator subsequently was related to the outcome measure in the predicted direction. This analysis is especially important for discovering unexpected relationships that may mask an overall intervention effect due to suppression effects [41].

## Results

**Figure 1:**
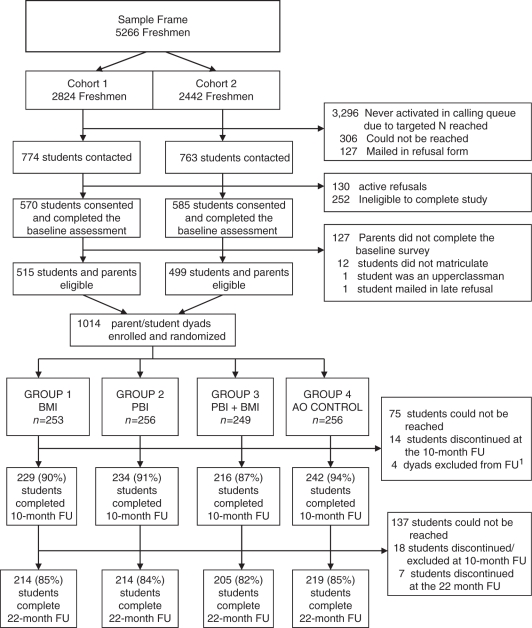
Student recruitment and retention AO, assessment only; BMI, brief motivational intervention; PBI, parent-based intervention; FU, follow-up. ^1^One parent–student dyad was excluded from follow-up due to death (unrelated to the study). Three dyads were removed because student participants began working at the survey center collecting data for this trial.

Successful recruitment of a representative sample of 1014 student–parent dyads was achieved across 2 cohort years with minimal refusal (Figure 1). Urn randomization produced equivalent groups at baseline in terms of demographic and primary outcome variables. The student sample was 57% (*n* = 580) female with a mean age of 18.4 years (SD = 0.41). The parent sample was 59% (*n* = 594) female. Retention of 90.8% (*n* = 921) of randomized students was achieved at the 10-month follow-up, and of 84% (*n* = 852) at the 22-month follow-up. Retention was significantly higher in the assessment-only group (94.5%) relative to the combined intervention group (86.8%) at 10 months. There were no significant differences in attrition by experimental group at 22 months and no baseline differences on any outcome variables between study completers and non-completers.

### Interventions

#### Intervention delivery and fidelity

Among students randomly assigned to receive the brief motivational intervention, 95% (*n* = 476) received the initial intervention (85% in-person, and 15% by mail), and 90% (*n* = 451) received the booster session (90% in-person and 10% by mail). Most students (≥92%) endorsed key components of the brief motivational intervention including high clinician rapport, empathy, and professionalism. Eighty-eight percent indicated feelings of enhanced self-efficacy.

Among parents randomly assigned to receive the parent intervention 89% (*n* = 448) completed the evaluation questionnaire by mail (*n* = 368) or telephone (*n* = 80). Approximately, 89% of responding parents reported being ‘very satisfied’ or ‘mostly satisfied’ with the handbook as a whole and reading ‘most’ or ‘all’ of the material. The handbook chapters were rated as useful, interesting, and understandable by approximately 84% of parents.

### Data collection and outcomes

Student data reflected a wide range of drinking behavior across all time points. As anticipated, data for the primary outcomes contained a large number of zero values (e.g., non-drinkers) and a large proportion of students engaging in HED and/or consequences. At baseline, 28% (*n* = 281) of students reported abstaining from alcohol for at least the past year, decreasing to 17% (*n* = 154) at the 10-month follow-up, and 13% (*n* = 112) at the 22-month follow-up (percentages adjusted for attrition). Approximately, half of the baseline sample (51%, *n* = 517) reported no instances of HED in the past month, and the mean number of consequences experienced in the past 3 months was 5.39 (SD = 7.67).

As reported elsewhere [42], the brief motivational intervention significantly reduced the onset of HED and alcohol-related consequences at 10 and 22 months. However, the observed effects were small and the parent-based intervention did not reduce onset or growth of HED or consequences. Evidence for the combined intervention effects was limited to alcohol-related consequences, with no effect observed for the combined intervention on HED. In terms of mediation, we found a consistent indirect brief motivational intervention effect through descriptive norms on both onset and growth in HED and consequences, but no evidence in support of hypothesized parent-based intervention mediators.

## Limitations and lessons learned

While the Transitions Project was successful in many respects, there are several limitations of this trial. In attempting to evaluate potential explanations for null and modest intervention effects, several design issues initially considered in the planning of this trial, re-emerged. Chief among these include questions were: (1) did assessment reactivity take place among parents and/or students? (2) did our assessment schedule miss critical short-term intervention effects?; and (3) were our interventions delivered with fidelity? We also have considered how these questions could have been answered with alternative experimental designs and study procedures.

The potential for assessment reactivity to attenuate or mask intervention effects is a serious and common problem in clinical research and is especially problematic when the size of intervention effects is modest [40,43]. We believe our assessment protocol, which included an in-depth assessment of process-related variables at baseline, may have potentially masked parent-based intervention effects by motivating control-group parents to engage in behavior that may have reduced alcohol use among students. Previous research has sought to avoid this issue through the use of a post-test only comparison design [30]. We opted for a pre-test post-test design in order to better model change over time. However, to disentangle intervention and assessment effects, an alternative study design is necessary, such as the Solomon Four Group Design [44] which crosses two intervention arms with baseline assessment (Yes/No). However, in most large clinical trials this approach is prohibitively expensive and impractical. Newer, more efficient, methods for evaluating assessment reactivity are available which involve including ‘planned missingness’ in the assessment design [45].

Another aspect of our study design that deserves consideration is assessment timing. In the social sciences assessment, timing is often dictated by convenience or tradition rather than empirically based expectations regarding intervention effect periods [46]. We reviewed relevant research and found that intervention efficacy was well documented through a 6-month follow-up period for brief motivational interventions, with several exceptions [47,48]. For this reason, we decided to focus on longer term outcome assessments to determine whether these interventions could produce lasting change. The use of lengthy assessments coupled with generous and increasing participation incentives enabled strong tests of the primary and secondary aims but used a large proportion of study resources, thus limiting our ability to afford a short-term assessment. Upon completion of this trial, we believe our null findings reflect a failure to capture critical periods of short-term intervention effects that decayed over time. Therefore, we recommend using three follow-up time points in combination with a baseline assessment to detect long-term and short-term, potentially transient, intervention effects and to model non-linear (e.g., quadratic, piecewise) effects.

Our lack of objective intervention fidelity measures is an additional limitation of this trial. Subjective measures of intervention fidelity employed by this study indicated the interventions were delivered as intended, but a lack of objective measures limits the strength of our inferences. Consistent with prior research [30], our parent handbook evaluation asked parents whether they read and understood the intervention materials. Parents may have provided socially desirable responses; the non-anonymous nature of the assessment may have exacerbated this effect. Similarly, participants in the brief motivational intervention were asked about clinician qualities and intervention components. Although their responses were collected anonymously after sessions, the possibility of subjective biases cannot be ruled out. In fact, our measures of intervention fidelity were relatively high and invariant across all categories for both interventions suggesting the possibility of social desirability and ceiling effects.

Ideally, future studies should examine the quality of intervention delivery using objective means, such as delivering the handbook online and tracking parent access. In terms of the brief motivational intervention, future trials should audio or video-record sessions and have them coded by at least two independent reviewers. Detailed, more objective procedures for assessing brief motivational intervention fidelity are available and increasingly expected in clinical trials [49,50]. Nonetheless, our supervision approach and the brief motivational intervention training we employed have been used in previous randomized controlled trials that obtained very good estimates of motivational interviewing consistency [10,51,52].

## Conclusions

In conclusion, the Transitions Project had many strengths, most notably the use of a factorial design capable of testing unique and combined effects of two potentially complementary interventions and the implementation of novel data-analytic techniques uniquely suited to our data. Limitations included our inability to explicate the extent to which assessment reactivity or the length of our follow-up interval may help explain the lack of support for some study hypotheses.

In terms of study successes, a large sample of students and parents were recruited prior to college matriculation. Consistent with other research works, the use of monetary ballooning incentives and a highly trained survey center was likely integral to recruitment and long-term retention success [31,53,54]. Intervention administration and participation were also high. Abstainers and drinkers were willing to attend in-person interventions and parents were willing to read mailed intervention materials and return evaluation forms. These participation and retention rates should encourage future researchers who attempt to use similar techniques and study similar populations. By including abstainers in our study, we were able to examine intervention effects among an at-risk but understudied group. The data complexity, the abstainers introduced (e.g., a high-zero count) was handled using latent growth curve modeling as an alternative to traditional data transformation. As published examples of two-part latent growth curve modeling are limited, interested readers are directed to our outcomes paper [42] and other published studies using this technique [29,38].

In summary, the choice of experimental and quasi-experimental designs in randomized controlled trials is a complex, multi-faceted endeavor with inevitable tradeoffs. We hope that consideration of the lessons we have learned and presented will benefit those who undertake similar research in the future.
